# Examination of residency program websites for the use of gendered language and imagery

**DOI:** 10.1186/s12909-023-04677-4

**Published:** 2023-09-26

**Authors:** Catherine E. Read, Jovanna A. Tracz, Nour Mhaimeed, Rylie N. Mainville, Carrie A. Elzie

**Affiliations:** 1 School of Medicine, Eastern Virginia Medical School, Norfolk, VA 23507 USA; 2 Department of Cell Systems and Anatomy/Medical Education, University of Texas Health Science Center at San Antonio, 78229 San Antonio, TX USA

**Keywords:** Graduate medical education, Academic medicine, Women in medicine, Women in surgery, Subspecialty choice, Gender

## Abstract

**Background:**

Significant disparity in gender distribution exists among medical specialties. Residency program websites are a main source of preliminary program information for candidates, and website content may influence a prospective applicant’s sense of belongingness within a particular program. Given the importance of the residency program website as a recruiting tool, this study sought to examine and compare the presence of gendered language and imagery on residency program websites across various specialties.

**Methods:**

A list of words considered masculine or feminine was used to evaluate residency program websites of the two most male-dominated specialties (orthopedic and thoracic surgery), female-dominated specialties (pediatrics and obstetrics and gynecology), and gender-balanced specialties (dermatology and family medicine) in the United States in 2022. Forty-five residency programs were randomly selected from each specialty across different regions of the US, with the exception of thoracic surgery of which there are only 33 programs. Masculine and feminine words were evaluated using a parsing and scraping program. Representation of female and male-presenting team members in photos on program websites was also evaluated.

**Results:**

Masculine wording occurred more frequently in male-dominated specialties compared to gender-balanced (p = 0.0030), but not female-dominated specialties (p = 0.2199). Feminine language was used more frequently in female-dominated compared to male dominated fields (p = 0.0022), but not gender balanced (p = 0.0909). The ratio of masculine-to-feminine words used was significantly higher in male-dominated specialties compared to both gender-balanced (p < 0.0001) and female-dominated specialties. (p < 0.0001). There was an average of 1, 7, and 10 female-presenting residency team members pictured on each male-dominated, gender balanced, and female-dominated specialty RPW respectively, with significantly more female-presenting team members pictured in the photographs on female-dominated specialty websites when compared to male-dominated and gender-balanced specialty websites (p < 0.0001, p = 0.014).

**Conclusions:**

The use of gendered language and female representation in photographs varies significantly across specialties and is directly correlated with gender representation within the specialty. Given that students’ perceptions of specialty programs may be affected by the use of language and photos on residency program websites, programs should carefully consider the language and pictures depicted on their program websites.

**Supplementary Information:**

The online version contains supplementary material available at 10.1186/s12909-023-04677-4.

## Background

As a traditionally male-dominated field, gender diversity in medicine continues to be a critical topic. Although progress has been made with more female medical graduates, women still only represent 36% of all US physicians. A greater discrepancy exists in gender distribution among surgical specialties, including orthopedic surgery (5.8% female), thoracic surgery (8% female), and neurosurgery (9.3% fem﻿ale) [1]. However, studies investigating the factors that contribute to these discrepancies have typically focused on promoting interest in male-dominated fields, rather than the sources shaping female medical student decision-making when applying to male-dominated residency programs [2–10]. Other studies have identified specific concerns prospective female residency applicants have regarding male-dominated specialties; however, these studies did not uncover the sources which influence these perceptions [11–13]. While all students cited location, program reputation, and proximity to family as contributing factors in program rank decisions, women and under-represented minority students tend to assess and weigh additional factors related to culture, inclusion, and diversity more than others [14]. In a survey of interviewees about the perception of gender and diversity representation among program faculty and residents, the perception of diversity and representation positively influenced program ranking by female and under-represented minority medical students [15]. In addition, many candidates rely heavily on residency program websites (RPW) when deciding where to apply, interview, and rank [16, 17]. Since RPW have become a nearly universal part of recruitment efforts, factors embedded in websites that might influence the perception of gender diversity warrant further investigation. Although a recent study assessed elements of diversity on general surgery program websites using eight factors, [18] no factors addressed language.

Language is an important factor in the creation and reinforcement of an individual’s identity, particularly in the context of in-group and out-group interactions, associations, and biases [19]. While gender differences in language use likely reflect a complex combination of social goals, situational demands, and socialization, the origins of these differences are beyond the scope of this paper. Within medicine, linguistic gender biases have most recently been explored in letters of recommendation. It has been shown that language use differs based on the gender of whom one is writing about, as women are described more frequently using communal traits (i.e., adjectives that describe a concern for the welfare of others), such as peacemaking behavior, or descriptors of nurturing, gentleness or kindness. In contrast, men are more commonly described using agentic traits, which are stereotypically leadership-oriented, such as confidence, assertiveness, and influence [20]. Such examples have been shown to be present in evaluations of medical students [21] and residents [22], as well as letters of recommendations. A recent systematic review found that women applicants were more likely to be described using communal adjectives (“delightful” or “compassionate”), while men applicants were more likely to be described using agentic adjectives (“leader” or “exceptional”) [23]. An over-reliance of communal adjectives may result in the perception that women lack leadership qualities [24–26]. Letters for women applicants also had more frequent mentions of personal appearance [27, 28] and personal life details [20, 29]. Often recommendation letters include doubt-raising language (e.g., ‘hedging’ language, or veiled criticism) or irrelevant gendered descriptions (e.g., mentions of physical appearance), which may further undermine an applicant’s suitability for the role [20, 28] (Trix; Turrentine). For example, in recommendation letters for surgery resident applications, letter for male candidates contained more achievement words (performance, career, leadership, and knowledge), while caring words (care, time, patients, and support) were used more often for female applicants [28, 30, 31]. However, other reports indicate that language in letters of recommendation were similar between men and women applicants, as well as men and women letter writers [32]. When present, these descriptive gendered differences may have a negative impact on the applicant during the selection process often disadvantaging women applicants.

Many women working in academic medicine describe feeling marginalized, as they perceive themselves to be outsiders, reporting feelings of isolation and not belonging [33]. Word choice and selective forms of address can contribute to this through implication of a gender hierarchy and support of an “in group” bias [20, 34, 35]. Even prior to taking a job, the presence of gendered language has been identified as a significant contributor to female applicants’ perceptions of potential jobs, their sense of belongingness in a field, and their desire to pursue a given career [36]. Further evidence has demonstrated that gendered wording in job advertisements contributes to sustained gender inequality in the workforce [37–40]. Drawing from this literature, we reasoned that gendered wording may emerge within residency program information materials as a subtle mechanism of maintaining gender inequality by dissuading women from more male-dominated fields. Thus, this project sought to identify if gendered language was present on RPW across a variety of specialties including male-dominated, female-dominated and gender-balanced fields. The study also aimed to correlate the gender of physicians featured in pictures on RPW, as differential depiction of physicians in different specialties may also contribute to the decision to apply to a particular residency program or rank one program higher than another [15, 40–42].

## Methods

A list of United States (US) medical specialties was obtained from the Association of American Medical Colleges which comprised 47 specialties. Each specialty was categorized as male-dominated, female-dominated, or gender balanced using the 2020 Physician Specialty Data Report: Active Physicians by Sex and Specialty, 2019 [1]. Only specialties with corresponding residency programs were analyzed. Dual specialty programs, such as internal medicine/pediatrics, were not included in analysis and respective specialties were stratified independently. The two most male-dominated specialties (orthopedic surgery, 5.8% female; thoracic surgery, 8% female), female-dominated specialties (pediatrics, 64.3% female; obstetrics and gynecology, 58.9% female), and gender balanced specialties (dermatology, 51% female; family medicine, 41.3% female) were selected for comparison. The programs were chosen simply by ranking the programs with the highest percentages of males and females and the top two respective programs were chosen for analysis. For gender neutral programs, the two programs with the closest ratio of males to females were chosen.

A list of residency programs was obtained from the Electronic Residency Application Service [43]. To account for geographic diversity, five residency programs from each of the 9 US census divisions were selected for each specialty, for a total of 45 residency programs per specialty analyzed, except for thoracic surgery of which only 33 integrated residency programs exist. Residency programs were selected from each census region via a random number generator. The regions used were New England, Middle Atlantic, South Atlantic, East North Central, East South Central, West North Central, West South Central, Mountain, and Pacific as defined by the US Census Bureau. Programs outside of the continental US and Hawaii were excluded.

Of the selected RPW for analysis, the content analyzed consisted of the “About the Program” page or a “Letter from the director” obtained in 2022. A list of words associated with feminine or masculine connotations was generated from previous literature on the effects of gendered language in hiring and on the use of gendered language in evaluations of medical students (Supplemental Table 1) [21, 36]. This list was originally generated from published lists of agentic and communal words (e.g., individualistic, competitive, committed, supportive) [44, 45] and masculine and feminine trait words (e.g., ambitious, assertive, compassionate, understanding) [46–48]. A scraping and parsing program built in Python, version 3.10.5 (Python Software Foundation, Fredericksburg, Virginia) was implemented to analyze the selected pages of each RPW for gendered language use. The accuracy of the software program was verified by comparing parsed data to manual analyses of three websites with 100% congruence between the manual and computer analyses. For the 25 residency program websites which contained security functionality that did not permit use of the scraping and parsing program, analysis was conducted manually. Conjugations of base words were included in analysis. If an individual word or a conjugation of the word was used more than once, each use was recorded as an additional instance. The number of masculine and feminine words was calculated for each website and averaged for each specialty. The ratios of masculine to feminine words and feminine words to masculine words were also calculated for each RPW and averaged for specialties. Differences in the use of masculine and feminine wording were compared between male-dominated and female-dominated specialties, between male-dominated and gender-balanced specialties, and between female-dominated and gender-balanced specialties using One-Way ANOVA and a Tukey’s Multiple Comparison test (95% Confidence Interval) calculated with Prism Software Version 8.0.0 for Windows (GraphPad Software, San Diego, CA, USA).

**Fig. 1:**
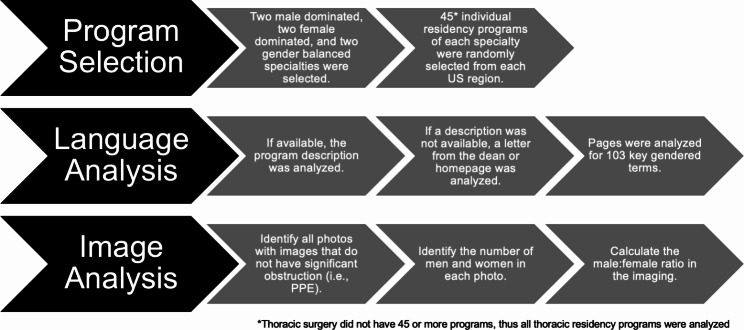
Method used in the analysis of residency program websites for gendered language and imagery

If images were present on the webpage , the number of male-presenting and female-presenting individuals was recorded manually by two of the authors (CR and JT) independently; no discrepancies were present. All photos on the website were included in the analysis. If there was significant obstruction by personal protective equipment that prevented clear determination, the individual was not analyzed. The average number of female-presenting individuals in website images was compared between male-dominated and female-dominated specialties, between male-dominated and gender-balanced specialties, and between female-dominated and gender-balanced specialties using One-Way ANOVA and a Tukey’s Multiple Comparison test (95% Confidence Interval) using Prism Software Version 8.0.0 for Windows (GraphPad Software, San Diego, CA, USA) (Fig. 1).

## Results

A total of 258 RPW were analyzed for both gendered wording and gendered imagery. There was significant variation of gendered language use across RPW. The number of masculine words ranged from 0 to 46; the number of feminine words ranged from 0 to 103. RPW for orthopedic surgery used an average of 4 masculine and 7 feminine words; thoracic surgery used an average of 7 masculine and 8 feminine words; dermatology used an average of 4 masculine and 8 feminine words; family medicine used an average of 6 masculine and 13 feminine words; pediatrics used an average of 6 masculine and 16 feminine words; and OB/GYN used an average of 4 masculine and 11 feminine words per website.

**Fig. 2:**
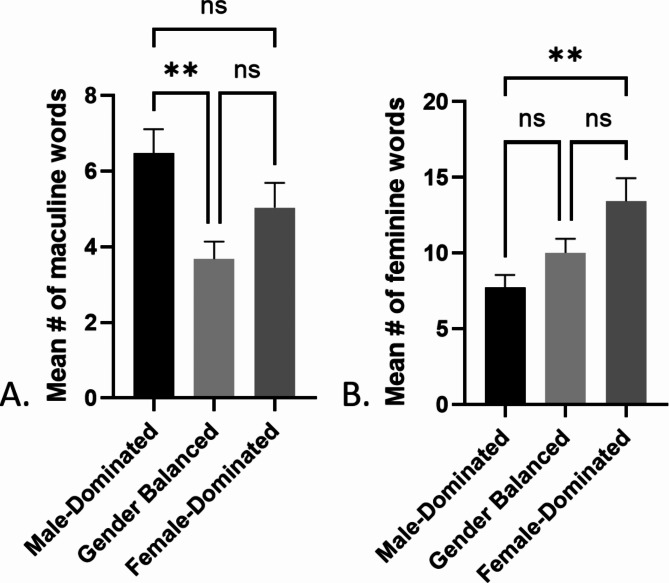
**A**. Mean number of masculine words on masculine-dominated, gender balanced, and female-dominated RPW. **B**. Mean number of feminine words on masculine-dominated, gender balanced, and female-dominated RPW (mean ± SEM, **p < 0.01)

A one-way ANOVA test revealed there was a significant difference in frequency of masculine words used in RPW between at least two groups (male-dominated, female-dominated, and gender-balanced specialties) (F(2,253)=[5.509], p = 0.0046). A posthoc Tukey’s test for multiple comparisons determined there was a significantly higher use of masculine words in male-dominated specialties when compared to the gender-balanced specialties (p = 0.0030, CI = 0.8109-4.790), but not when comparing the female-dominated specialties to either the male-dominated (p = 0.2044, CI=-0.5487-3.430) or gender-balanced specialties (p = 0.2199, CI=-3.282-0.5631) (Fig. 2). A one-way ANOVA test revealed that there was also a significant difference in frequency of feminine words used in RPW between at least two groups (male-dominated, female-dominated, and gender-balanced specialties) (F(2,253)=[5.930], p = 0.0030). A post-hoc Tukey’s comparison determined there was a significantly higher use of feminine words in female-dominated compared to male-dominated specialties (p = 0.0022, CI=-9.642- ^−^1.751) but not compared to gender-balanced specialties (p = 0.0909, CI=-7.218-0.4090). There was no difference between gender-balanced and male-dominated specialties use of feminine words (p = 0.3586, CI=-6.237-1.654) (Fig. 2).

**Fig. 3:**
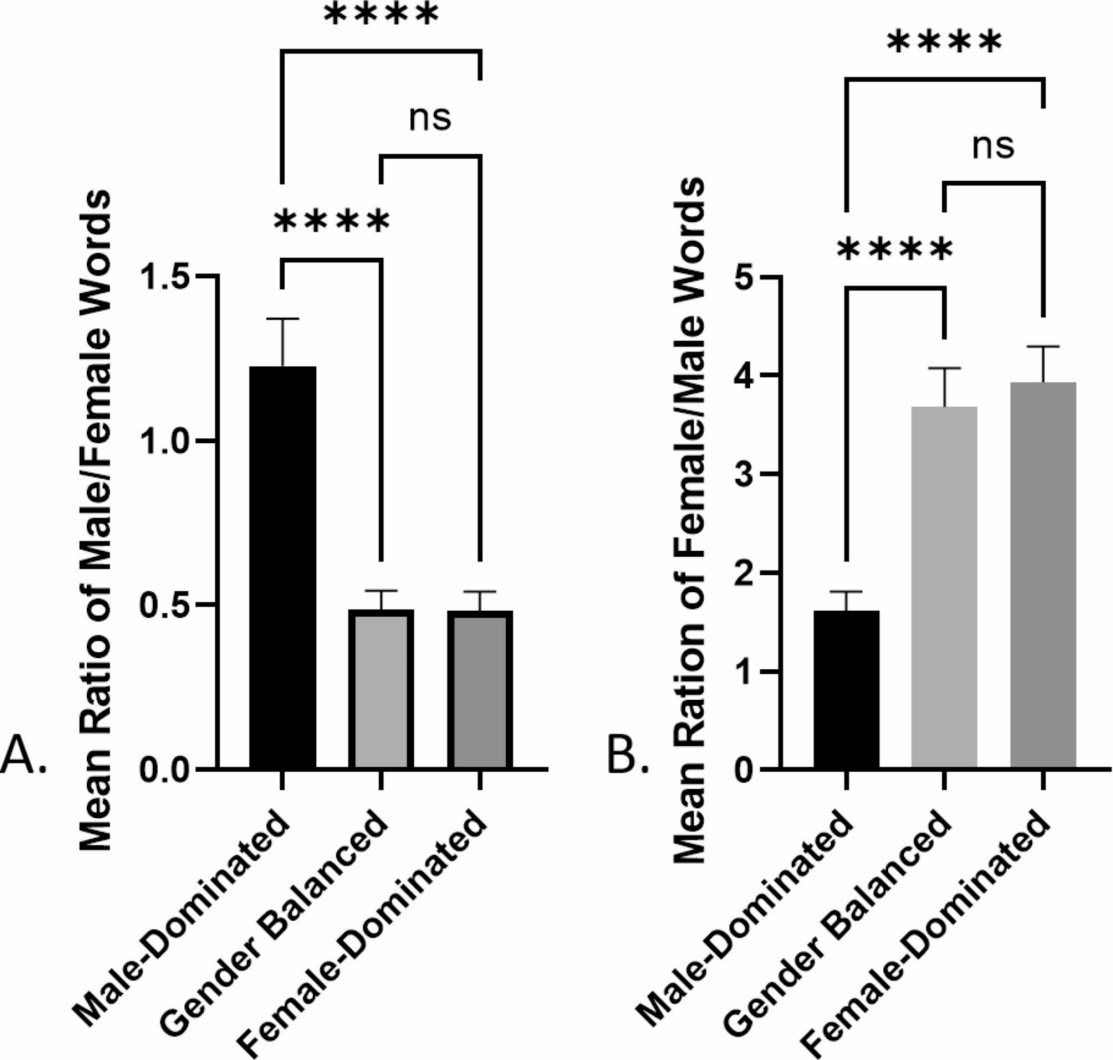
**A**. Mean ratio of masculine-to-feminine words on masculine-dominated, gender balanced, and female-dominated RPW. **B**. Mean ratio of feminine-to-masculine words on masculine-dominated, gender balanced, and female-dominated RPW (mean ± SEM, ****p < 0.0001)

To determine if there were differences between the average ratios of masculine-to-feminine words (# of masculine words divided by # of feminine words) on RPW, a one-way ANOVA was performed. There was a statistically significant difference in mean ratios between at least two groups (F(5, 253) = [21.34], p < 0.0001). To further delineate the differences between the specialties a Tukey’s HSD Test for multiple comparisons was performed. Male-dominated specialties had a significantly higher ratio of masculine to feminine words when compared to both gender-balanced (p < 0.0001, CI = 0.4359–1.048) and female-dominated (p < 0.0001, CI = 04388 − 1.052) specialties. There was no difference in the ratio of masculine to feminine words used in RPW between gender-balanced and female-dominated fields (p = 0.9995, CI= -0.2923-0.2999) (Fig. 3). This may be because the ratio of males to females in gender-balanced specialties compared with female-dominated specialties is more closely aligned than compared to the ratio in the male-dominated fields. When broken down by specialty, both male-dominated specialties (orthopedics and thoracic surgery) had a significantly higher ratio of masculine-to-feminine words when compared to the gender-balanced and female-dominated specialties. There were no differences reported between the two specialties within each gender category (orthopedics vs. thoracic surgery; dermatology vs. family medicine; or pediatrics vs. OB/GYN) (Supplemental Table 2).

A one-way ANOVA of the reverse ratio of feminine-to-masculine words (number of feminine words divided by number of masculine words) also showed a significant difference in mean ratios between at least two groups (male-dominated, female-dominated, and gender-balanced specialties) ((F(5, 253) = [13.68], p < 0.0001). A Tukey’s HSD Test for multiple comparisons showed a significant difference in ratio of feminine-to-masculine words between female-dominated and male-dominated specialties (p < 0.0001, CI=-3.456- -1.185) and between male-dominated and gender-balanced specialties (p < 0.0001, CI=-3.203- -0.9318). However, there was no difference in ratio of feminine-to-masculine words in RPW between female-dominated and gender-balanced fields (p = 0.8500, CI=-1.350-0.8447) (Fig. 3). Further analysis between specialties revealed that OB/GYN RPWs specifically had a significantly higher ratio of feminine-to-masculine words when compared to both male dominated specialties; pediatrics had a higher ratio compared only to orthopedics; family medicine used a greater ratio than both male dominated specialties. There were no differences in the ratio of feminine-to-masculine words used between the gender-balanced and female-dominated specialties, nor was a significant difference found between specialties within each category (Supplemental Table 3).

**Fig. 4:**
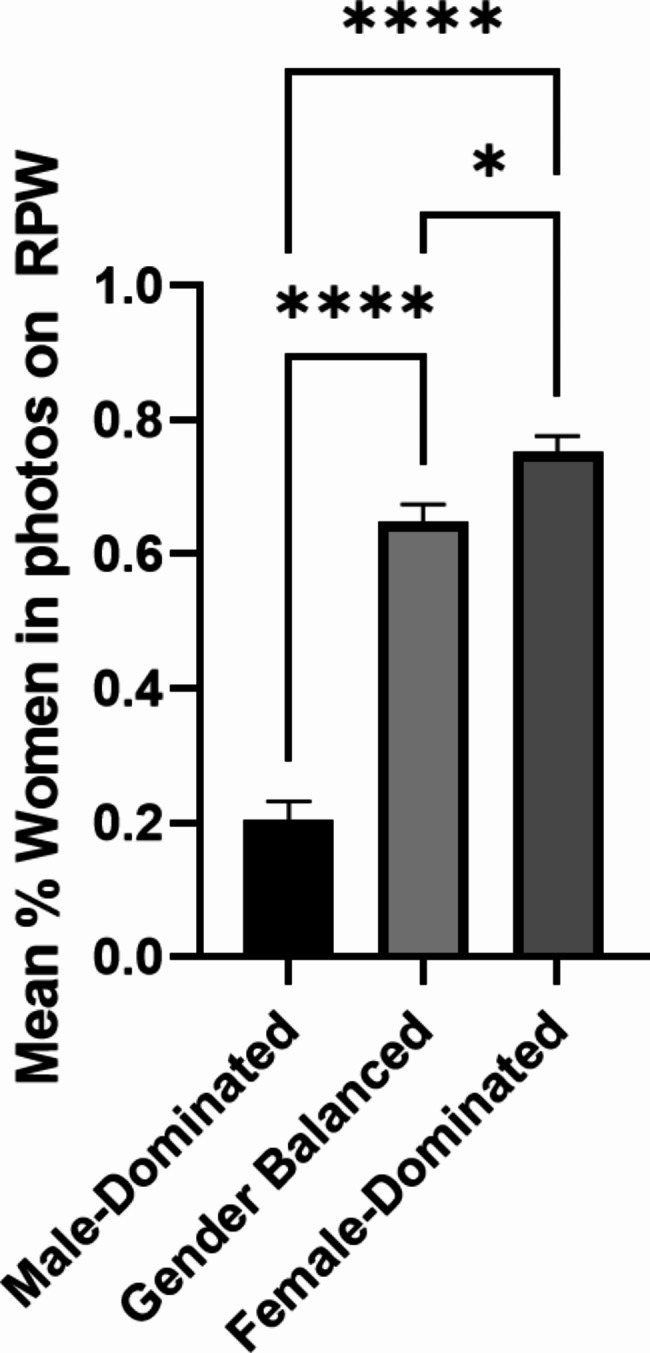
Mean percent of females represented compared to males in pictures on masculine-dominated, gender balanced, and female-dominated RPW (mean ± SEM, *p < 0.05, ****p < 0.0001)

There was an average of 1, 7, and 10 female-presenting residency team members pictured on each male-dominated, gender balanced, and female-dominated specialty RPW respectively, with significantly more female-presenting team members pictured in the photographs on female-dominated specialty websites when compared to male-dominated and gender-balanced specialty websites (p < 0.0001, p = 0.014). There were also significantly more female-presenting team members depicted in images on gender-balanced specialty websites when compared to male-dominated specialty websites (p = 0.0004) (Fig. 4).

## Discussion

To understand why women continue to be underrepresented in traditionally male-dominated fields, it may be beneficial to investigate the interplay between institutional-level factors, such as information portrayed on RPWs, and the perception of certain specialties. Evidence suggests that a sense of belongingness affects achievement motivation specifically and engagement within a domain more generally, and that belongingness can be signaled by cues in the work environment [49–51]. Indicators of belongingness, such as the use of gendered language and imagery on RPW, may contribute to a prospective applicant’s sense of belongingness within a particular program. While numerous recommendations for residency programs to improve their online presence to engage medical student applicants have been reported, none were specific to imagery or language [52]. However, data reported here indicates differences in the use of gendered language and imagery across RPW, which may influence medical student perceptions of various medical specialties.

The subtlety of gendered wording can directly affect the appeal of various jobs for different genders and therefore may be a contributor to inequality. Whereas gendered pronouns and other explicit references to the gender of a candidate can be detected by readers, gendered wording is comparatively veiled. In a previous study using a broad spectrum of job advertisements in which participants were given job descriptions with intentionally gendered wording, women reported greater anticipated belongingness within occupations that were more femininely than masculinely worded; however, they were unable to identify gendered language as the variable that was altered [36]. Women were also more affected by gendered language then men, especially with the impact on belongingness within the occupation [36]. This may also occur in medical students sense of belongness based on RPW, as data here showed higher numbers of masculine words used in male-dominated specialties compared to gender-balanced specialties and a higher ratio of masculine-to-feminine words compared to female-dominated specialties. In previous studies, women were more interested in male-dominated jobs when the advertisements were unbiased, making reference to both men and women as candidates, than when the advertisements made reference only to men [53]. High levels of masculine wording in job advertisements deterred women from those jobs. Further, women viewed masculine worded job advertisements as less gender diverse and less appealing (due to less belongingness, and not perceived skills), compared with jobs advertised with feminine wording [20, 36]. This is congruent with social role theory that posits men and women will find jobs described in language consistent with their own gender most appealing precisely because it signals they belong in that occupation [54]. Thus, minimizing the use of incidental masculine wording in RPWs may not only increase the number of women in male-dominated fields, but also lead to greater numbers of women seeking training in these specialties. Thus, we recommend all programs to take a critical look at the language used on their websites and other promotional materials to target diverse candidates and foster inclusion.

Images on RPW provide additional information about the workplace environment. In surveys of plastic surgery residents regarding the most important information on RPW, 90–95% of survey respondents included faculty profiles and current resident information as two of the most valued pieces of information on RPW [55]. Data reported here demonstrated a significant discrepancy in gender distribution among images displayed on RPW, which was also seen when comparing male-dominated to gender neutral specialties, as well as female-dominated to gender neutral specialties. Observations of women’s underrepresentation in an environment—and the anticipation that one would become part of the minority in the environment—may result in expectations of being negatively stereotyped, perceptions that the environment is inequitable to women, lower confidence about one’s ability, a lower sense of belongingness and less desire to apply [56–58]. Consequently, in a time when social distancing requirements and safety precautions have already drastically changed the path toward the residency match, improving website quality and content will be an increasingly essential recruitment, communication, and showcasing tool for residency programs.

### Limitations

Data reported here indicated disparities exist with respect to gendered language on RPWs using two dictionaries of gendered language. One of the two dictionaries was created for a broad spectrum of jobs and may not best represent terms deemed as gendered in the medical field specifically. The creation of a new dictionary vetted by medical students and specific to the medical field may provide a more accurate analysis. Additionally, the methodology of this study is insufficient to prove a cause-and-effect relationship between gendered language on RPW and gender disparity of residents. Data here and previously reported literature suggest that language and imagery may sway medical student residency application decisions, but does not demonstrate if it actually does or to what extent the influence may be. Future work will be aimed at manipulating the wording and imagery of RPW to determine how this may influence applicants’ rankings of programs. An additional limitation of this work was the examination of gender as a binary on RPW. The authors wholly acknowledge that gender is a spectrum and many individuals do not identify with binary descriptions. While our nascent work focused on male and female classifications, further work is needed to better explore gender nonconforming and nonbinary language rooted biases. We also acknowledge that external appearances do not always reflect gender identity especially transgender or gender nonbinary individuals who may feel the need to hide their identity to avoid discrimination [59]. Thus, our manual categorization of gender based on photos alone may not be accurate. Additionally our work focused on residency program websites which may not accurately reflect the current diversity of practicing physicians (non-trainees). However, the intention of this paper was to identify sources that may contribute to the disparity of female-identifying residents in traditionally male-dominated specialties and to suggest areas of improvement. It was not intended to exclude any parties based on gender identity or expression. It is our hope that through continued exploration of disparities in medicine and the improvement of representation, that better tools and systems will be developed and employed in future work that will capture the spectrum of gender diversity in a more comprehensive manner. Further, the findings of this work do not diminish the complexity of gender disparity in residency programs; however, it does indicate a rather direct and immediate area for improvement to counteract a broader gendered culture within medicine.

## Conclusions

This is the first study, to our knowledge, to investigate the use of gendered language and images on residency program websites in the continental United States. The data presented in this study demonstrate the existence disparity in the use of gendered language on RPW between male and female dominated medical specialties. As work continues towards improved gender equity amongst medical specialties, residency programs may benefit from using this data as a starting point to improve the language and imagery used on their websites. This understanding of potential language-rooted biases in the medical field can guide simple changes for increased workplace inclusivity. While our study focused on gender differences, it is worthwhile to investigate the impact of language on other aspects of diversity in medicine as well.

### Electronic supplementary material

Below is the link to the electronic supplementary material.

Supplementary Material 1

Supplementary Material 2

Supplementary Material 3

## Data Availability

The datasets used and/or analyzed during the current study are available from the corresponding author on reasonable request.

## Abbreviations

RPW: Residency program websites
