# Supplementary material for: Examination of residency program websites for the use of gendered language and imagery
Source: BMC Med Educ. 2023 Sep 26;23:697. doi: 10.1186/s12909-023-04677-4 (PMC10523617; doi:10.1186/s12909-023-04677-4)
Supplement: Supplementary file 1 — Supplementary Material 1 [file 12909_2023_4677_MOESM1_ESM.docx]

**Supplemental Data**

**Table 1: List of masculine and feminine words depicted in residency program websites.**

Dictionary of masculine and feminine words used to evaluate their occurrence on residency program websites. * indicates the words found on RPW.

**Masculine words:**

Active*

Acute*

Adventurous

Affable

Aggressive*

Ambitious*

Analytic*

Assertive

Athletic*

Autonomous*

Basic*

Boastful*

Certain*

Challenging*

Competitive*

Confident*

Courageous

Decisive*

Deep*

Determined

Dominant

Earnest

Easy-going

Forceful

General*

Good*

Great*

Greedy

Headstrong

Hierarchical

Hostile

Humble

Impulsive

Independent*

Individual*

Intellectual*

Leader*

Logical*

Objective*

Opinionated*

Outspoken

Persistent

Principled*

Reckless

Relaxed

Relevant*

Respectful*

Scientific*

Stubborn

Superior

Self-confident

Self-sufficient

Self-reliant

**Feminine words:**

Affectionate*

Caring*

Child-like

Cheerful

Communal*

Compassionate*

Comprehensive*

Connected*

Considerate*

Cooperative*

Delightful

Dependent*

Efficient*

Emotional

Empathetic*

Energetic*

Enthusiastic*

Fabulous

Flatterable

Gentle

Honest

Interpersonal*

Interdependent

Kind*

Kinship

Lovely

Loyal*

Modest

Nagging

Nurturing

Pleasant

Poised*

Polite

Quiet

Sensitive*

Social*

Submissive*

Supportive*

Sympathetic

Tender

Together*

Trusting

Understanding*

Warm

Welcome*

Whining

Wonderful*

Yield
