# Supplementary material for: Examination of residency program websites for the use of gendered language and imagery
Source: BMC Med Educ. 2023 Sep 26;23:697. doi: 10.1186/s12909-023-04677-4 (PMC10523617; doi:10.1186/s12909-023-04677-4)
Supplement: Supplementary file 2 — Supplementary Material 2 [file 12909_2023_4677_MOESM2_ESM.docx]

**Table 2. Comparison of the ratio of masculine to feminine words across specialties.**

Ratio of masculine to feminine words was compared between specialties using a Tukey’s multiple comparison test. Table represents p values for each comparison (*p<0.05, **p<0.01).

|  | Thoracic Surgery | Dermatology | Family Medicine | Pediatrics | Obstetrics  Gynecology |
| --- | --- | --- | --- | --- | --- |
| Orthopedics | 0.9260 | 0.0122* | <0.0001** | <0.0001** | 0.0002** |
| Thoracic Surgery | ------------- | 0.2862 | 0.0001** | 0.0051 | 0.0266* |
| Dermatology | ------------- | ------------- | 0.0997 | 0.5966 | 0.8958 |
| Family Medicine | ------------- | ------------- | ------------- | 0.9153 | 0.6404 |
| Pediatrics | ------------- | ------------- | ------------- | ------------- | 0.9946 |
