# Supplementary material for: Examination of residency program websites for the use of gendered language and imagery
Source: BMC Med Educ. 2023 Sep 26;23:697. doi: 10.1186/s12909-023-04677-4 (PMC10523617; doi:10.1186/s12909-023-04677-4)
Supplement: Supplementary file 3 — Supplementary Material 3 [file 12909_2023_4677_MOESM3_ESM.docx]

**Table 3. Comparison of the ratio of feminine to masculine words across specialties.**

Ratio of feminine to masculine words was compared between specialties using a Tukey’s multiple comparison test. Table represents p values for each comparison (*p<0.05, **p<0.01).

|  | Thoracic Surgery | Dermatology | Family Medicine | Pediatrics | Obstetrics  Gynecology |
| --- | --- | --- | --- | --- | --- |
| Orthopedics | 0.9995 | 0.5936 | <0.0001** | 0.0163* | 0.0004** |
| Thoracic Surgery | ------------- | 0.8559 | 0.0003** | 0.0856 | 0.0051** |
| Dermatology | ------------- | ------------- | 0.0087 | 0.5829 | 0.0923 |
| Family Medicine | ------------- | ------------- | ------------- | 0.4492 | 0.9688 |
| Pediatrics | ------------- | ------------- | ------------- | ------------- | 0.9085 |
